# Conjunctive BSA-Seq and BSR-Seq to Map the Genes of Yellow Leaf Mutations in Hot Peppers (*Capsicum annuum* L.)

**DOI:** 10.3390/genes15091115

**Published:** 2024-08-23

**Authors:** Guosheng Sun, Changwei Zhang, Xi Shan, Zhenchao Zhang, Wenlong Wang, Wenjun Lu, Zhongliang Dai, Liu E, Yaolong Wang, Zhihu Ma, Xilin Hou

**Affiliations:** 1 State Key Laboratory of Crop Genetics & Germplasm Enhancement and Utilization, Nanjing Agricultural University, Nanjing 210095, China; 2022404006@stu.njau.edu.cn (G.S.); changweizh@njau.edu.cn (C.Z.); 2023804298@stu.njau.edu.cn (W.W.); 2021104078@stu.njau.edu.cn (L.E.); 2022104074@stu.njau.edu.cn (Y.W.); 2 Zhenjiang Institute of Agricultural Sciences in Hilly Area of Jiangsu Province, 1# Hongjing Road, Jurong 212400, China; shanxi@jaas.ac.cn (X.S.); 20072803@jaas.ac.cn (Z.Z.); 2023104067@stu.njau.edu.cn (W.L.); daizhongliang@jaas.ac.cn (Z.D.)

**Keywords:** hot pepper, yellow leaf mutant, BSA-Seq, BSR-Seq, *CaKAS2*, *CaMPH2*

## Abstract

Yellow leaf mutations have been widely used to study the chloroplast structures, the pigment synthesis, the photosynthesis mechanisms and the chlorophyll biosynthesis pathways across various species. For this study, a spontaneous mutant with the yellow leaf color named 96-140YBM was employed to explore the primary genetic elements that lead to the variations in the leaf color of hot peppers. To identify the pathways and genes associated with yellow leaf phenotypes, we applied sequencing-based Bulked Segregant Analysis (BSA-Seq) combined with BSR-Seq. We identified 4167 differentially expressed genes (DEGs) in the mutant pool compared with the wild-type pool. The results indicated that DEGs were involved in zeatin biosynthesis, plant hormone signal transduction, signal transduction mechanisms, post-translational modification and protein turnover. A total of 437 candidates were identified by the BSA-Seq, while the BSR-Seq pinpointed four candidate regions in chromosomes 8 and 9, containing 222 candidate genes. Additionally, the combination of BSA-Seq and BSR-Seq showed that there were 113 overlapping candidate genes between the two methods, among which 8 common candidates have been previously reported to be related to the development of chloroplasts, the photomorphogenesis and chlorophyll formation of plant chloroplasts and chlorophyll biogenesis. qRT-PCR analysis of the 8 common candidates showed higher expression levels in the mutant pool compared with the wild-type pool. Among the overlapping candidates, the DEG analysis showed that the *CaKAS2* and *CaMPH2* genes were down-regulated in the mutant pool compared to the wild type, suggesting that these genes may be key contributors to the yellow leaf phenotype of 96-140YBM. This research will deepen our understanding of the genetic basis of leaf color formation and provide valuable information for the breeding of hot peppers with diverse leaf colors.

## 1. Introduction

Hot peppers (*Capsicum annuum* L.) are rich in vitamin C, capsaicin and capsaicin-like substances, making them valuable in various fields, such as food, medicine, industry and chemistry. As an important economic crop, the market size of chili peppers is growing worldwide [1]. The chloroplast is an essential organelle responsible for photosynthesis, using chlorophyll pigments to absorb red and blue light while reflecting green light [2]. Hot peppers have various kinds of leaf color mutations, with yellow leaf mutations being the most common. Leaf etiolation mutants typically exhibit yellow-green leaves during the seedling stage or throughout the entire growth period [3,4], and yellow leaf mutation is a high-quality germplasm resource. Leveraging these features can accelerate the breeding process, such as developing plants that are more resistant to light stress. Yellow leaf mutations hold significant value in variety breeding and improvement, as they can simplify the process of seed purity identification. It has been reported that leaf etiolation mutants exist in crops such as cotton, maize, rice and cabbage [5,6].

In the past, leaf color mutants were often considered detrimental, as they could affect leaf color formation, leading to decreased photosynthetic efficiency and reduced dry matter yield. Since Granick used the chlorotic mutant W_5_ of Chlorella vulgaris to study the synthesis mechanism of chlorophyll in 1948 [7], leaf color mutants have been widely utilized in basic research, including genetic patterns, pigment synthesis, photosynthesis, cell structure and development [7,8,9,10,11]. As a common mutation, plant leaf color variation frequently occurs during the seedling stage. Obtaining leaf color mutants is of vital importance for the study of plant leaf growth and development. Due to the significant importance of leaf color research, this key plant trait has garnered the attention of many researchers. It has been established that the coloration of leaf color mutants results from a combination of genetic and environmental factors. Leaf color is mainly influenced by chloroplast development and chlorophyll metabolism [12]. As a photosynthetic pigment, chlorophyll can absorb light and transfer light energy to the photosynthetic system, which plays a critical role in plant development [13]. Thus, genetic factors are the fundamental cause of leaf color variation. Currently, research on the regulatory genes of leaf color mutants are primarily focused on a few model plants and vegetable crops [14].

The mechanism of leaf etiolation is due to the decomposition of chlorophyll or the disturbance of other pigment synthase genes [13,14,15,16,17,18,19,20,21,22,23]. Several yellow-green leaf genes are associated with components photosystem I, photosystem II, photosynthetic electron transport and F-type ATPase [24,25]. Through transcriptome sequencing, researchers have identified that the key genes involved in chlorophyll biosynthesis are chlorophyll a/b-binding proteins, light-harvesting chlorophyll a/b-binding proteins and chlorophyll a/b-binding protein precursors [26]. In wheat, mutations at the cn-A1, cn-B1 and cn-D1 loci lead to reductions in the expression levels of chlorophyll a/b complex II [27,28,29]. In rice, the ygl1 mutant exhibits yellow-green leaves due to the reduced expression of the *YGL1* gene, which leads to decreased chlorophyll synthase activity [30]. The *chl1* and *chl9* genes, isolated from rice chlorina mutants, are involved in chlorophyll synthesis by participating in the formation of Mg-chelatase [31]. In cucumbers, the *CscpFtsY* and *CsSRP43* genes have been identified as major regulators of leaf yellowing [32]. Due to the ease of observation and stability of the yellow leaf color, it has been widely used as a genetic marker in various crops, including rice [33], wheat [34], soybeans [35], rapeseed [36], cucumbers [37], cantaloupes [38] and tomatoes [39]. Long-term manual selection has narrowed the variation types in peppers, limiting the genetic improvement of certain special traits [40].

In this study, a recessive mutant, 96-140YBM, was isolated from the wild type 96-140. This mutant has yellow new leaves that turn green when they mature. Yellow leaf pepper mutant ‘96-140YBM’ was crossed with the wild type ‘96-140’, and the F_2_ segregation progeny were generated for BSA and BSR-Seq. Our results will enhance our understanding of the genetic and transcriptional changes in the yellow leaf pepper mutant. 

## 2. Materials and Methods

### 2.1. Plant Materials and Development Conditions

**Figure 1:**
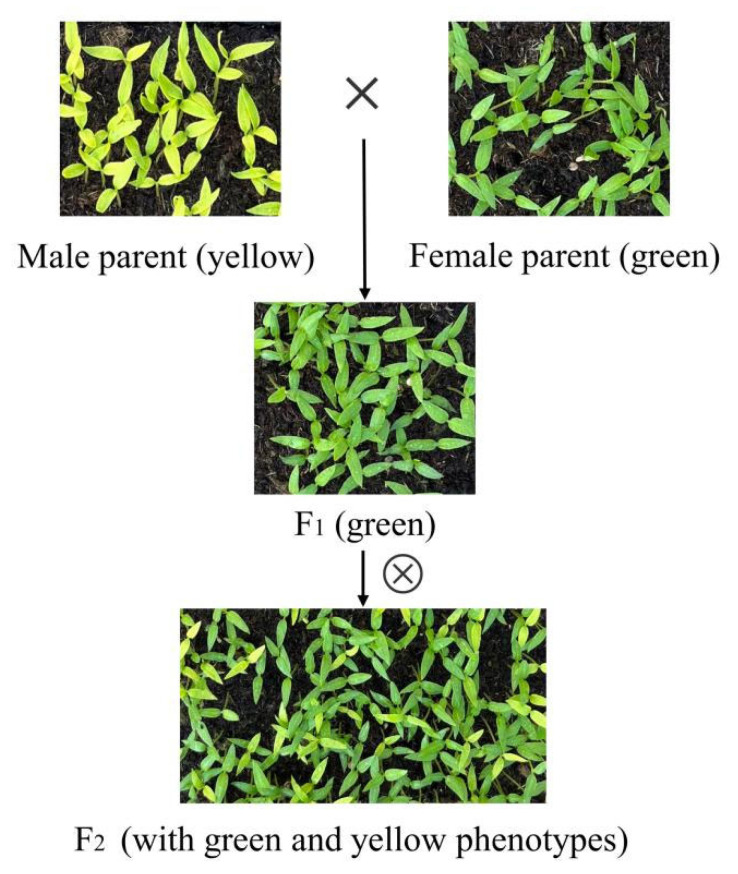
The emergence of F_2_-segregating offspring with the contrary leaf color trait.

The leaf-yellowing mutant ‘96-140YBM’ and the wild type ‘96-140’ were crossed to produce F_1_ seedlings (Figure 1). The ‘96-140YBM’ type served as the maternal parent, while ‘96-140’ was used as the paternal parent. F_2_-segregating offspring were produced through the self-pollination of F_1_ plants. Over several years, leaves from the parent plants and 100 individuals with contrasting leaf color traits in the F_2_ progeny were collected (50 plants with yellow leaves and 50 plants with green leaves). New yellow leaves and old green leaves were collected to construct high-throughput sequencing libraries. Samples of 50 mg were collected from every plant, with three biological replicates established. For the real-time PCR analysis, 2 individual plants exhibiting extreme green and yellow leaf traits were selected from the F_2_ progeny.

### 2.2. Establishment of the Illumina Library for Bulked Segregant Analysis

For the BSA-Seq, we assembled 4 DNA pools: 2 parental lines and 2 F_2_-segregating offspring. The parent bulks, R01 and R02, were generated from ‘96-140’ and ‘96-140YBM’, respectively. The R03 bulk was obtained from 50 F_2_ plants with green leaves, while the R04 bulk was similarly created using 50 F_2_ plants with yellow leaves. The four sequencing libraries were sequenced on the Illumina HiSeqTM2500 platform.

### 2.3. Investigation of BSA-Seq Data

After base calling analysis, the raw files generated by high-throughput sequencing were converted into raw sequencing reads. Subsequently, using BWA (Burrows Wheeler Aligner) V2-2.2.1 software [41], high-quality clean readings were compared to *Capsicum annuum* L. (Pepper Zunla 1 Ref-v2.0) [41]. SAMtools was used to eliminate duplicates and mitigate the impact of PCR duplication [42]. The local realignment and base recalibration were carried out using GATK (Genome Analysis Toolkit) [15]. SnpEff V 5.0 software was utilized for the annotation of the identified SNPs and indels [43]. LOESS regression fitting was performed on the Δ (SNP index) values on the same chromosome to obtain the relevant threshold. The average *p*-value ≤ 0.05.

### 2.4. Construction of cDNA Library in BSR-Seq

In order to analyze BSR-Seq, 4 cDNA bulks were established (T01, T02, T03 and T04). The total RNA was extracted from fresh leaves. Subsequently, transcriptome sequencing was performed on the Illumina HiSeq™ 2500 platform (Illumina, San Diego, CA, USA).

### 2.5. Investigation of BSR-Seq Data

We compared the filtered readings to the reference genome *Capsicum annuum* L. and used edgeR V3.28.1 software to identify the DEGs between two sets of BSR Seq blocks (T01 vs. T02, T03 vs. T04), with dispersion set to 0.1. The Benjamini and Hochberg method was used to adjust the *p*-value to calculate the false discovery rate (FDR) [44]. The standard setting for determining DEGs was |log_2_ (fold-change)| ≥ 1 and FDR < 0.01. GATK was used to detect SNPs and small indels and perform sliding-window analysis on the association analysis of Δ (SNP-index).

### 2.6. qRT-PCR Validation

The total RNA was extracted using the total RNA extraction kit (Tiangen Biotech, Nanjing, China), digested with DNase I and reverse-transcribed into cDNA. We took 2 μL of diluted cDNA as a template, according to SYBR^®^ Premix ExTaq (Takara, Kyoto, Japan), to detect these gene expression levels. 

## 3. Results

### 3.1. Bulked Segregant Analysis

#### 3.1.1. Investigation of Sequencing Data of Four DNA Bulks

In total, 731.91 million paired-end reads were produced, with approximately 125.45 million and 129.33 million reads generated for R01 and R02 (Table 1) and 235.76 million and 241.37 million reads for R03 and R04, respectively. Following the alignment of clean reads to the *Capsicum annuum* reference genome assembly, using Pepper Zunla 1 Ref_v2.0, the coverage was 69X for the parental bulks and 97X for the F_2_ progeny bulks. 

#### 3.1.2. Association Analysis Regarding BSA-Seq

**Figure 2:**
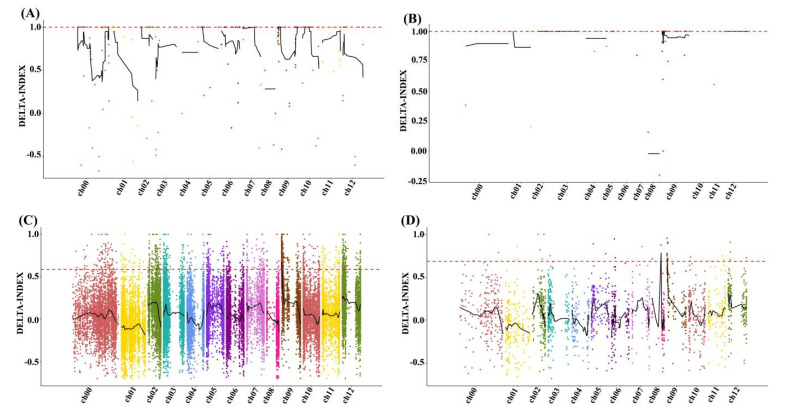
The Δ (SNP-index) values of chromosomes were calculated to identify the candidate regions associated with the yellow leaf in hot peppers based on the BSA-Seq and BSR-Seq. (**A**) Through the association analysis of particular SNPs between the T03 and T04 bulks using BSA-Seq, the candidate regions were anchored. (**B**) Through the association analysis of particular small indels between R03 and R04, the candidate regions were anchored. (**C**) Through the association analysis of particular SNPs between the T03 and T04 bulks using BSR-Seq, the candidate regions were anchored. (**D**) Through the association analysis of particular small indels between T03 and T04, the candidate regions were anchored.

In total, 490212 SNPs and 60310 small indels were identified between R03 and R04. Subsequently, 5141 SNPs and 946 small indels that had different genotypes in R03 and R04 were chosen for association analysis (coverage depth > 5X). Additionally, 13 regions accountable for the yellow leaf were anchored on Chr 02, Chr 05, Chr 07, Chr 09 and Chr 10, having a total length of 17.96 M (Figure 2A), and 9 candidate regions related to the yellow leaf were anchored on chromosomes Chr 01, Chr 02, Chr 03, Chr 05 and Chr 09, with a total length of 0.9 M (Figure 2B).

A total of 437 candidate genes were included in the two methods (Figure 2C,D, Table 2 and Table S1). In the KOG database, the candidate genes were enriched in signal transduction mechanisms and general function predictions only (Figure S1).

### 3.2. Bulked Segregant RNA-Seq Analysis

#### 3.2.1. Analysis of Sequencing Data for Four cDNA Bulks

The T01 and T02 bulks produced 20.53 million and 22.73 million clean reads, respectively, while the T03 and T04 bulks generated 59.03 million and 55.92 million clean reads, respectively. The clean reads were aligned with the reference hot pepper (Pepper Zunla 1 Ref_v2.0), and the alignment efficiency was at least 89.74% (Table 3). 

**Figure 3:**
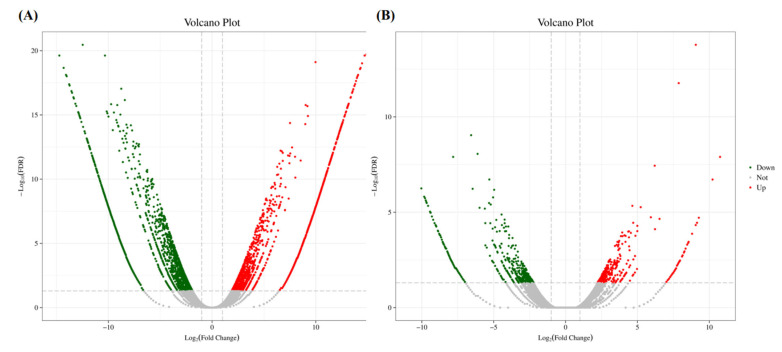
Volcano plots of the DEGs between two set pairs of cDNA sequencing bulks in BSR-Seq analysis. The green points represent the down-regulated genes; the red points represent the up-regulated genes. (**A**) T01 vs. T02. (**B**) T03 vs. T04.

A total of 4169 genes with DEGs were identified between the T01 and T02 bulks, between which 1804 genes were up-regulated and 2365 were down-regulated significantly (Figure 3A). Additionally, 929 DEGs were identified between the T03 and T04 bulks; there were 372 up-regulated genes and 557 down-regulated genes (Figure 3B).

#### 3.2.2. The Comparison of the Gene Expression Files of Two Set Pairs of cDNA Sequencing Bulks

**Figure 4:**
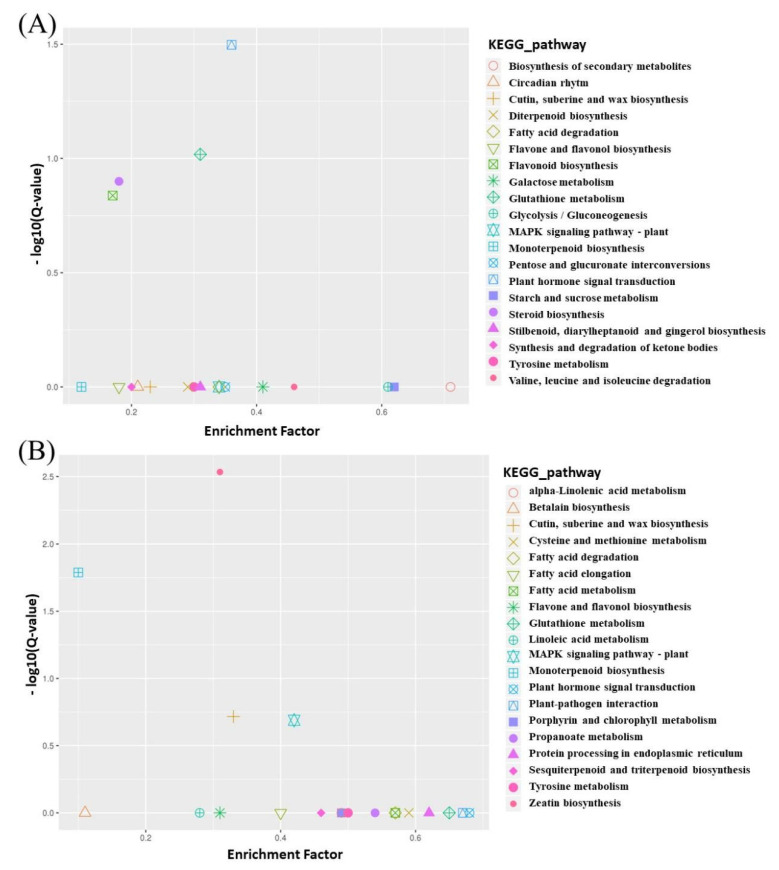
Graph of KEGG pathway analysis of DEGs between two set pairs of cDNA sequencing bulks. (**A**) T01 vs. T02. (**B**) T03 vs. T04.

As a consequence, the DEGs were assigned to three major GO terms. For T01 and T02, 351 DEGs were distributed in 112 KEGG pathways. The zeatin biosynthesis and monoterpenoid biosynthesis were significantly enriched (Figure 4A). For the T03 and T04 bulks, out of 69 KEGG pathways, there were 105 DEGs. The plant hormone signal transduction was significantly enriched (Figure 4B). 

The KOG function classification analysis showed that the DEGs were enriched in 21 pathways, including signal transduction mechanisms, post-translational modification, chaperones, protein turnover and secondary metabolite biosynthesis, transport and catabolism, et al. (Figure S2). 

#### 3.2.3. Association Analysis of BSR-Seq Data

**Figure 5:**
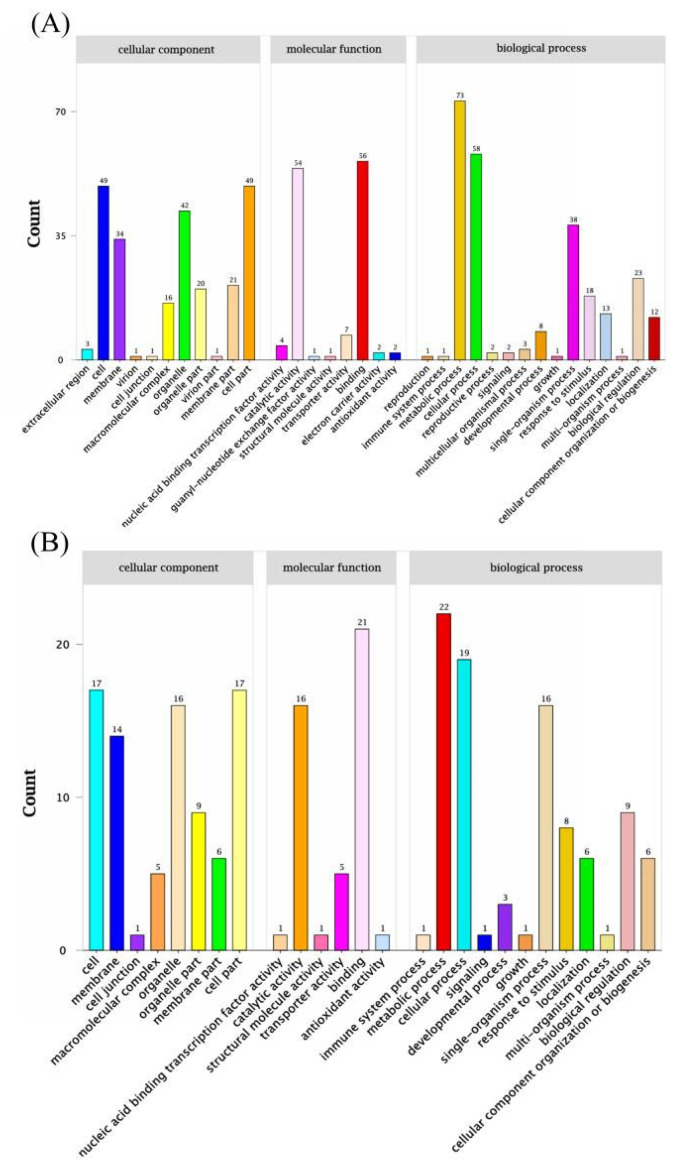
GO enrichment analysis of candidate genes identified by association analysis of BSR-Seq. (**A**) Candidate genes identified by the association analysis of specific SNPs between the T03 and T04 bulks using BSR-Seq. (**B**) Candidate genes identified by the association analysis of specific small indels between the T03 and T04 bulks.

Association analysis was conducted on 40793 SNPs and 2679 indels with significant differences (coverage depth > 5X) between bulks T03 and T04. Through association analysis of specific SNPs, two candidate regions were anchored on Chr09, containing 163 genes (Figure 5, Table 4 and Table S2), while the other two regions were determined through association analysis of specific indels, containing 60 genes (Table 4, Table 5 and Table S2). GO classification analysis showed that in the molecular functional category, these were mainly enriched in catalytic, binding and transport protein activity. In the cellular component category, the top five enriched items were the cell, cell part, organelle, organelle part and membrane (Figure 5A,B). The KOG classification analysis indicated that the candidate genes were enriched in general function prediction and signal transduction mechanisms (Figure S3). 

### 3.3. Morphological Genetic Analysis and Candidate Genes of Yellow Leaves

There were significant differences in leaf color between ‘96-140YBM’ and ‘96-140’, which could be directly observed. F_1_ plants from a cross between ‘96-140YBM’ and ‘96-140’ displayed green leaves. When the F_1_ plants were self-fertilized, the F_2_ plants exhibited yellow leaves and green leaves (Figure 1, Table S3). A total of 302 F_2_ plants were planted, and the leaf color was observed. Among these plants, 82 had yellow leaves and 220 had green leaves. This corresponds to a separation ratio of three to one. These results indicate that the formation of hot pepper yellow leaves was controlled by dominant genes, consistently with previous research findings [45]. 

Among the candidate genes identified by BSA-Seq, 113 genes were identified through the SNP index association analysis of BSR-Seq (Table 5 and Table S3). Many factors will affect the color of the plant leaves, like the development of chloroplasts, composition and content of chlorophyll, carotenoids and anthocyanins, and environmental factors. Therefore, we identified eight candidate genes that were related to the development of chloroplasts, the photomorphogenesis and chlorophyll formation of plant chloroplasts and chlorophyll biogenesis, including *CaMPH2* (*Capana09g000032*), *CaMGD2* (*Capana09g000045*), *CaAMK2* (*Capana09g000061*), *CaLAPA2* (*Capana09g000125*), *CaKAS2* (*Capana09g000140*), *CaCPN21* (*Capana09g000141*), *CaCRTL-E-1* (*Capana09g000177*), and chlorophyll A/B binding protein gene (*Capana09g000146*). Additionally, a number of candidate genes were related to the plant hormone signaling pathway, and there were three candidate genes in the ethylene pathway, including *CaWIN1* (*Capana09g000023*), *CaAP2L1* (*Capana09g000106*), *CaERF017* (*Capana09g000142*) and seven zeatin o-glucosyl transferase family gene *CaZOG1* (*Capana09g000111-116*, -*134*, -*136*), that have also been identified. 

We compared the 113 common candidates obtained by BSA-Seq and BSR-Seq with the DEGs between two set pairs of cDNA sequencing bulks (T01 vs. T02, T03 vs. T04) to hunt common candidates among them, which included two up-regulated genes, *CaKAS2* (*Capana09g000140*) and *CaMPH2* (*Capana09g000032*), and one down-regulated gene, *CaCKX3* (*Capana09g000187*). The up-regulated gene *CaKAS2* was notated as plastidic β-ketoacyl-ACP synthase II, the up-regulated gene *CaMPH2* was notated as green lineage-specific thylakoid lumen protein and the down-regulated gene *CaCKX3* was notated as cytokinin dehydrogenase 3.

### 3.4. qRT-PCR Validation of the Expression Pattern of Candidates in Hot Peppers

**Figure 6:**
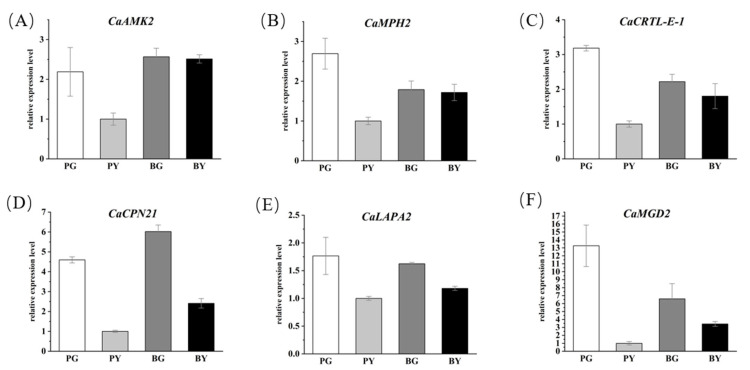
qRT-qPCR validation of the expression levels of six common candidates from combined BSA-Seq and BSR-Seq. (**A**–**F**) The relative expression levels of six genes, *CaAMK2* (*Capana09g000061*), *CaMPH2* (*Capana09g000032*), *CaCRTL-E-1* (*Capana09g000177*), *CaCPN21* (*Capana09g000141*), *CaLAPA2* (*Capana09g000125*) and *CaMGD2* (*Capana09g000045*), in the leaves of four different plants. PG: male parent (96-140); PY: female parent (96-140 YBM); BG: green leaf-type plant of F_2_ segregation bulks; BY: yellow leaf-type plant of F_2_ segregation bulks.

The expression patterns of the six common candidates were characterized using qRT-PCR and were identified by the combination of BSA-Seq and BSR-Seq. The qPCR analysis results showed that the expression levels of six common candidates were markedly higher in the green wild leaf of the male parent (96-140) compared with the yellow mutant leaf of the female parent (96-140 YBM) and also showed higher expression levels in the green leaf-type plant of the F_2_ segregation bulks (Figure 6). 

## 4. Discussion

As a rapid and efficient tool for genetic mapping, BSA has been frequently used to identify specific loci associated with target phenotypes in various plant species, such as rice [46], maize [47], watermelon [48], Chinese cabbage [49], wheat [50], soybeans [51] and peppers [52]. BSA approaches detect target loci at the genetic level [53]. As a modification of BSA, BSR tools have been exploited not only for gene mapping but also for providing global patterns of gene expressions combined with transcriptional profiles [54]. In this study, a total of 437 candidate genes located on chromosomes Chr02, Chr03, Chr05 and Chr09 were identified through BSA-Seq association analysis. Four candidate regions, located on chromosomes Chr08 and Chr09, were detected by BSR-Seq, including 222 genes. There were 113 candidate genes identified between the two methods. Functional annotation of the DEGs and candidate genes revealed that the most enriched pathways included signal transduction mechanisms, plant hormone signal transduction, zeatin biosynthesis, monoterpenoid biosynthesis, general function prediction and energy production and conversion. Collaborative filtering with the DEG analysis of BSR-Seq identified two common candidates, *CaKAS2* (*Capana09g000140*) and *CaMPH2* (*Capana09g000032*), that exhibited significantly different expression patterns between the mutant pool and the non-mutant pool.

The pathways were related to chloroplasts and chlorophyll, including porphyrin and chlorophyll metabolism, carotenoid biosynthesis, photosynthesis and carbon fixation in photosynthetic organisms [45]. Through the combined BSA-Seq and BSR-Seq, we identified eight candidate genes associated with chloroplast development, photomorphogenesis and chlorophyll formation, including photosystem II repair factor gene *CaMPH2* (*Capana09g000032*), chlorophyll a-b binding protein gene *CaCAB* (*Capana09g000146*), monogalactosyldiacylglycerol synthase gene *CaMGD2* (*Capana09g000045*), chloroplast gene *CaCPN21* (*Capana09g000141*), adenosine monophosphate kinase gene *CaAMK2* (*Capana09g000061*), plastidic β-ketoacyl-ACP synthase II *CaKAS2* (*Capana09g000140*), lycopene epsilon cyclase gene *CaCRTL-E-1* (*Capana09g000177*) and leucine aminopeptidase 2 gene *CaLAPA2* (*Capana09g000125*). The analysis of the BSR-Seq data supports the notion that causal genes are often down-regulated in the mutant pool compared to the wild types [54]. Therefore, it is rational to speculate that *CaKAS2* and *CaMPH2* may play crucial roles in the positive regulation of leaf greening.

In plants, the de novo biosynthesis of fatty acids is catalyzed by fatty acid synthase (FAS) [41]. Among these, KAS enzymes play a crucial role in carbon chain elongation [42]. There were three classes of KASs reported in various species, KASI, KASII and KASIII, with KASII specifically involved in the condensation of palmitoyl-ACP (16:0-ACP) to stearoyl-ACP (18:0-ACP) [43,55]. KASII is a key enzyme that determines the ratio of 16-carbon (C16) and 18-carbon (C18) fatty acids in plant cells [44]. The 16:0-ACP and 18:0-ACP fatty acids are involved in multiple biological processes, including the production of essential glycerides and phospholipids for cellular signaling and the formation of very-long-chain fatty acids (VLCFAs) critical for plant development and the conversion of plant hormones involved in stress responses, such as jasmonic acid [56,57]. In *Arabidopsis*, the fab1 mutant, which harbors a Leu337Phe substitution in the *AtKAS2* gene, exhibits a significant reduction in KAS II enzyme activity compared to the wild type [55]. At low temperatures, this mutant showed decreased photosynthetic efficiency, accompanied by degradation of thylakoids and chloroplasts, and a reduction in chloroplast glycerolipids and chlorophyll content [58]. Previous studies have indicated that KAS2 is crucial for the transition of embryos from the globular to the heart stage in *Arabidopsis* [59]. We speculate that the *CaKAS2* gene might be involved in fatty acid biosynthesis, and its reduced expression in the mutant pepper plant (96-140YBM) could impact various biological processes, including cellular signaling, chloroplast development and photosynthesis.

In *Arabidopsis*, MPH2 has been identified as a lumenal protein with high confidence [60,61]. The rapid repair of PSII and strong phosphorylation of PSII reaction center proteins contribute to the higher photosynthetic capacity of highland barley under high light, making it well-adapted to stress environments compared to other Triticeae crops such as wheat, triticale and barley [62]. The tomato lumenal protein MPH2 is considered an ortholog of the Arabidopsis protein [60]. In environments with combined cold and drought stress, the expression level of the tomato MPH2 homologous gene is drastically reduced [63,64,65]. In this study, the common gene *CaMPH2* was identified through genetic and transcriptional analysis and showed lower expression levels in the mutant pool. qRT-PCR results indicated that *CaMPH2* expression was higher in wild-type plants compared with mutant plants (Figure 5B), consistently with the DEG analysis from the BSR-Seq.

## Figures and Tables

**Table 1:** The summarizing of sequencing data and the alignment results of the BSA-Seq.

Bulk	Clean Reads	Clean Data	Q30 (%)	Genome Coverage 10× (%)	Average Depth (×)	SNP Number	Alignment Efficiency (%)
R01	125,450,705	37,635,211,500	91.67	68.5	21.3478	7,131,298	99.13
R02	129,325,381	38,797,614,300	92.67	68.91	21.6186	7,464,055	99.41
R03	235,759,304	70,727,791,200	93.14	96.82	33.7568	10,429,419	99.21
R04	241,371,002	72,411,300,600	92.9	96.58	46.74	10,407,663	99.16

**Table 2:** The identified candidate genomic regions based on the association analysis of BSA-Seq.

Method	Chromosome	Start	End	Size (M)	Genes
SNP index	2	15,717,687	15,817,687	0.1	4
	2	3,098,963	3,718,171	0.62	2
	2	7,488,745	7,588,745	0.1	1
	5	2,844,929	2,944,929	0.1	7
	7	6,169,571	6,731,315	0.56	2
	9	18,288,580	20,398,403	2.11	31
	9	22,093,862	23,040,848	0.95	8
	9	227,478,236	227,578,236	0.1	3
	9	2,6013,861	26,524,148	0.51	4
	9	4,828,484	15,906,110	11.08	324
	9	98,520	1,575,124	1.48	59
	10	50,118,507	50,271,184	0.15	1
	10	96,017,825	96,117,825	0.1	1
Small indel index	1	15,142,615	15,242,615	0.1	3
	2	113,098,665	113,198,665	0.1	1
	2	162,781,799	162,881,799	0.1	5
	2	93,723,980	93,823,980	0.1	1
	3	12,350,622	12,450,622	0.1	8
	3	204,823,413	204,923,413	0.1	2
	3	35,380,979	35,480,979	0.1	6
	5	34,832,217	34,932,217	0.1	1
	9	102,543	202,543	0.1	2

**Table 3:** The summarizing of sequencing data and the alignment results of the BSR-Seq.

Bulk	Clean Reads	Clean Data	Q30 (%)	SNP Number	Alignment Efficiency (%)
T01	20,530,863	6,159,258,900	91.79	115,475	89.74
T02	22,736,055	6,820,816,500	91.83	122,203	91.53
T03	59,034,487	17,710,346,100	92.13	168,598	91.69
T04	55,912,808	16,773,842,400	92.21	163,342	91.71

**Table 4:** Candidate regions identified by association analysis of BSR-Seq.

Method	Chromosome	Start	End	Size (M)	Genes
SNP index	9	3,520,729	6,351,360	2.83	99
	9	82,366	2,105,241	2.02	64
Indel index	8	112,304,151	112,404,151	0.10	2
	9	147,534	1,565,785	1.42	58

**Table 5:** Candidate genomic regions identified by association analysis of BSA-Seq and BSR-Seq data.

Method	Chromosome	Start	End	Size (M)	Genes
SNP index	9	98,520	1,575,124	1.48	44
	9	3,520,729	6,351,360	2.83	68
Indel index	9	147,534	202,543	0.05	1

## Data Availability

The original contributions presented in the study are included in the article/Supplementary Material, further inquiries can be directed to the corresponding authors.

## Supplementary Materials

The following are available online at https://www.mdpi.com/article/10.3390/genes15091115/s1, Figure S1: KOG function classification analysis of candidate genes identified by the association analysis of BSA-Seq, Figure S2: KOG function classification analysis of DEGs between four cDNA sequencing bulks. (A) T01 vs. T02. (B) T03 vs. T04, Figure S3: KOG function classification analysis of candidate genes identified by the association analysis of BSR-Seq, Table S1: BSA select region gene annotation, Table S2: BSR select region gene annotation, Table S3: Morphological genetic analysis and candidate genes of yellow leaves.
