# Supplementary figures and images for: Conjunctive BSA-Seq and BSR-Seq to Map the Genes of Yellow Leaf Mutations in Hot Peppers (Capsicum annuum L.)
Source: Genes (Basel). 2024 Aug 23;15(9):1115. doi: 10.3390/genes15091115 (PMC11430990; doi:10.3390/genes15091115)

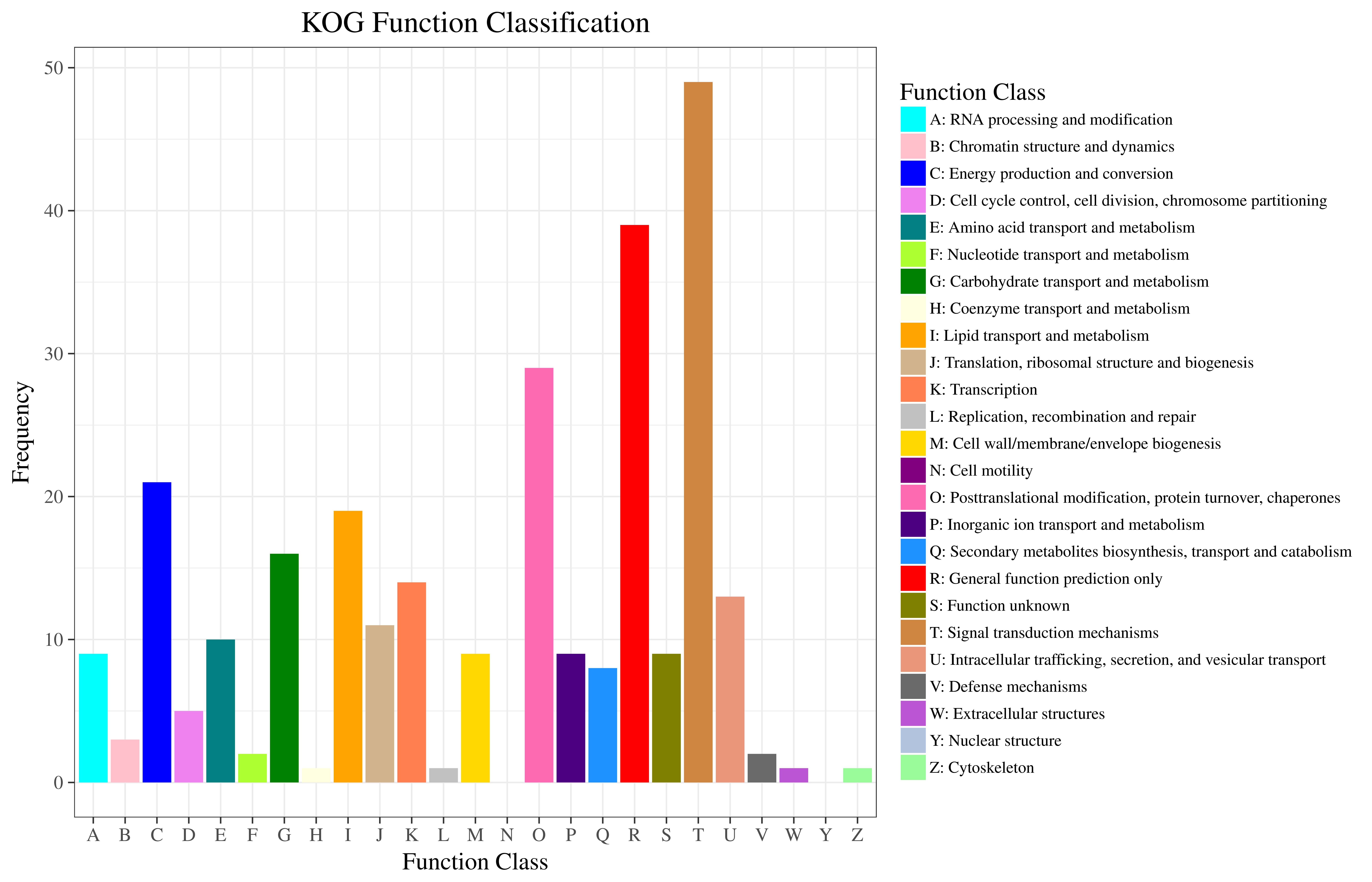

Supplement: Supplementary file 1 [file genes-15-01115-s001.zip › Supplementary Materials/Figure S1 KOG function classification analysis of candidate genes identified by the association analysis of BSA-Seq.jpg]

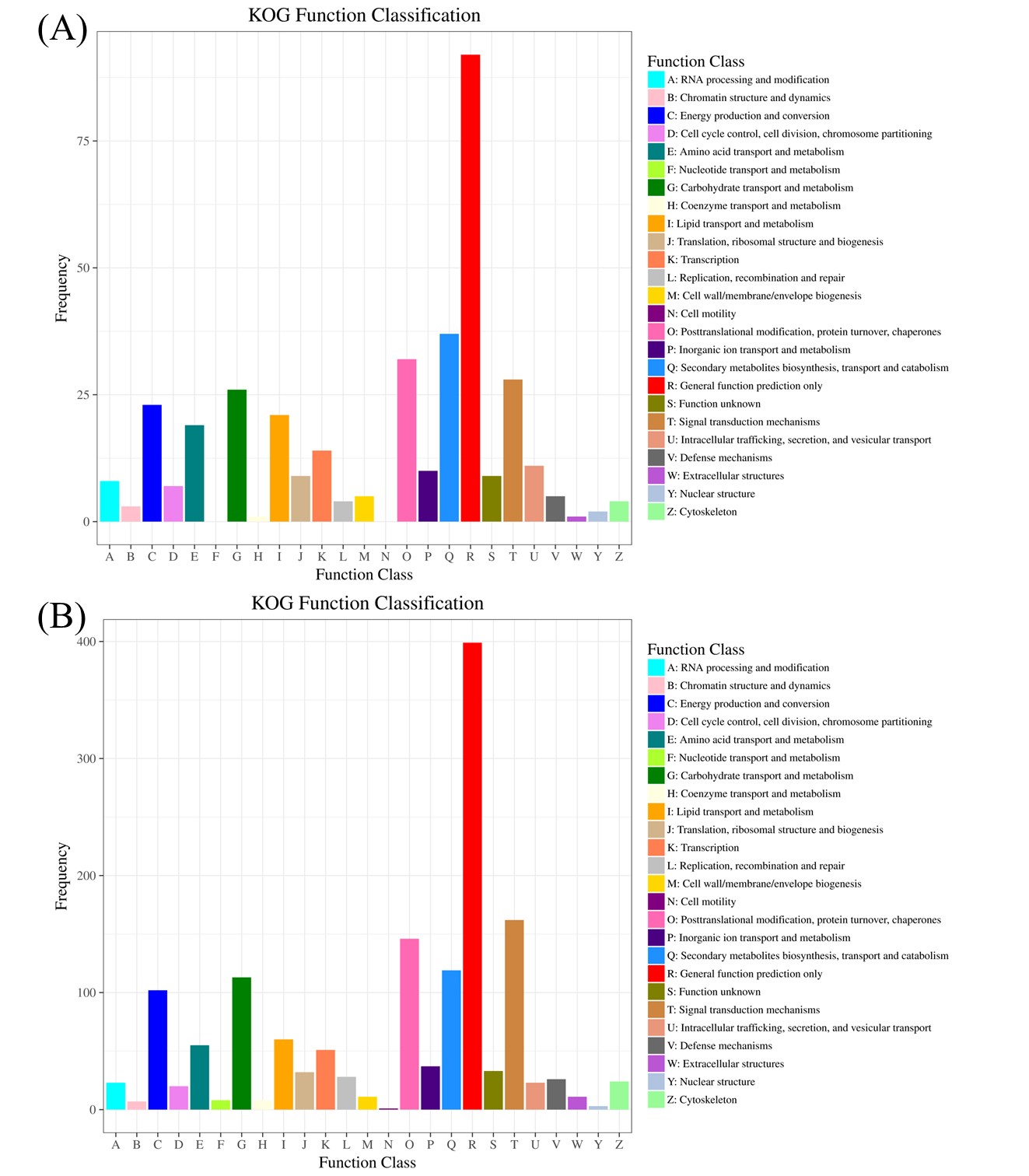

Supplement: Supplementary file 1 [file genes-15-01115-s001.zip › Supplementary Materials/Figure S2 KOG function classification analysis of differentially expression genes (DEGs) between four cDNA sequencing bulks. (A) T01 VS. T02. (B) T03 VS.jpg]

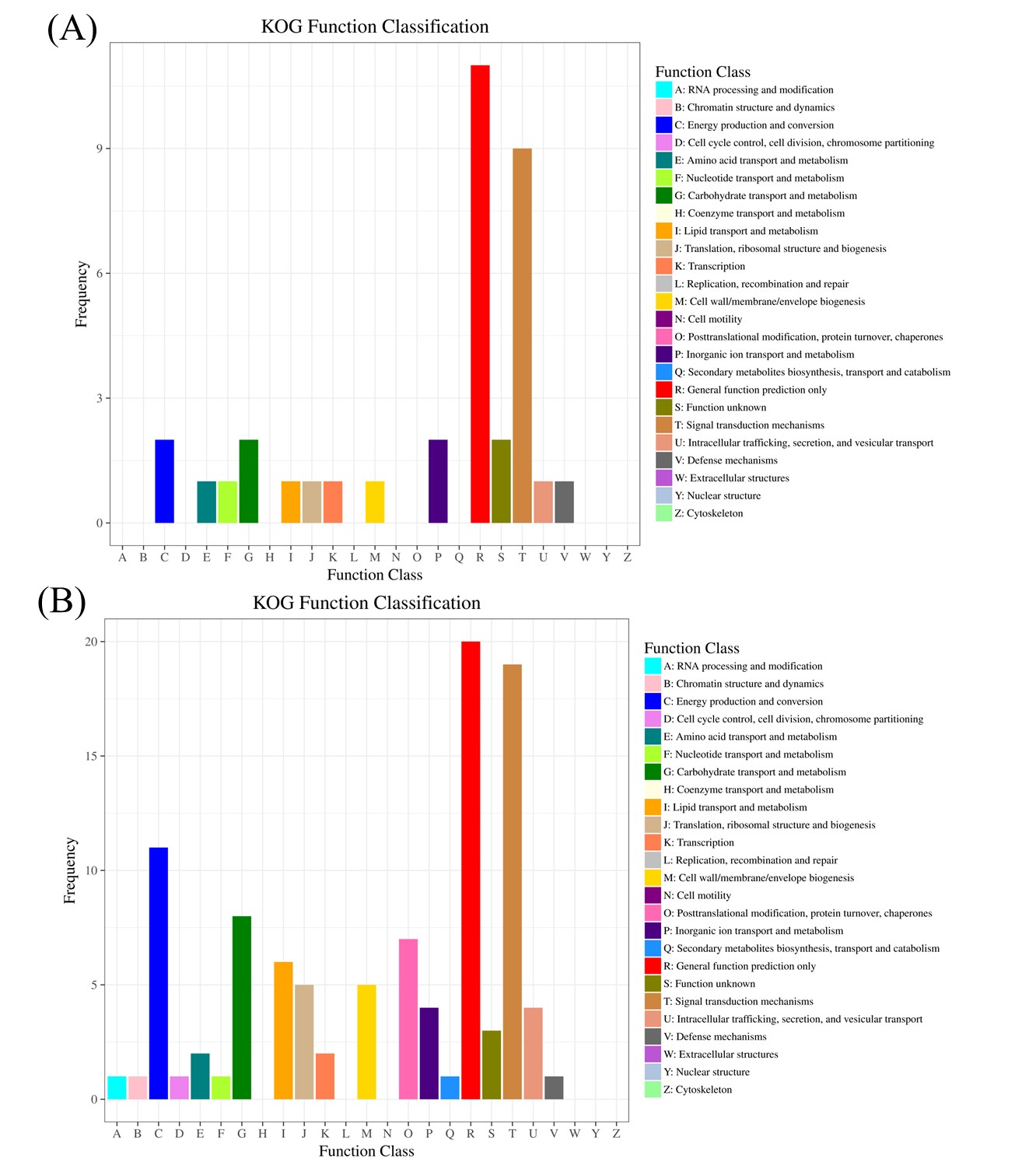

Supplement: Supplementary file 1 [file genes-15-01115-s001.zip › Supplementary Materials/Figure S3 KOG function classification analysis of candidate genes identified by the association analysis of BSR-Seq.jpg]
